# AI-driven deep CNN approach for multi-label pathology classification using chest X-Rays

**DOI:** 10.7717/peerj-cs.495

**Published:** 2021-04-20

**Authors:** Saleh Albahli, Hafiz Tayyab Rauf, Abdulelah Algosaibi, Valentina Emilia Balas

**Affiliations:** 1 Department of Information Technology, College of Computer Science, Qassim University, Buraydah, Saudi Arabia; 2 Centre for Smart Systems, AI and Cybersecurity, Staffordshire University, stoke on Trent, United Kingdom; 3 Department of Computer Science, King Faisal University, Hofuf, Saudi Arabia; 4 Department of Automation and Applied Informatics, Aurel Vlaicu University of Arad, Arad, Romania

**Keywords:** Image classification, Chest diseases, InceptionResNetV2, Pathology

## Abstract

Artificial intelligence (AI) has played a significant role in image analysis and feature extraction, applied to detect and diagnose a wide range of chest-related diseases. Although several researchers have used current state-of-the-art approaches and have produced impressive chest-related clinical outcomes, specific techniques may not contribute many advantages if one type of disease is detected without the rest being identified. Those who tried to identify multiple chest-related diseases were ineffective due to insufficient data and the available data not being balanced. This research provides a significant contribution to the healthcare industry and the research community by proposing a synthetic data augmentation in three deep Convolutional Neural Networks (CNNs) architectures for the detection of 14 chest-related diseases. The employed models are DenseNet121, InceptionResNetV2, and ResNet152V2; after training and validation, an average ROC-AUC score of 0.80 was obtained competitive as compared to the previous models that were trained for multi-class classification to detect anomalies in x-ray images. This research illustrates how the proposed model practices state-of-the-art deep neural networks to classify 14 chest-related diseases with better accuracy.

## Introduction

Medical X-rays, short for X-radiation, are a way to look to visible light in classical physics but with higher energy hits the body  (Panwar et al., 2020). X-ray is employed to generate images of tissues and structures inside the body; these include bones, chest, teeth, and so on  Rajaraman & Antani (2020). X-rays are handy diagnostic tools used for several decades by specialists to detect fractures, certain tumors, pneumonia, dental problems, and others. In advanced cases, CT (Computed Tomography) can produce a series of body images that are later assembled into a three-dimensional X-ray image processed by computer. However, the standard X-ray is faster, easier, cheaper, and less harmful than the CT scan  (Rajaraman & Antani, 2020).

For most cases, X-rays are not enough for the radiologist to carry out their diagnosis; they usually require CT scans to confirm the diseases  (Hashmi et al., 2020). Diseases with quick progression might even require multiple CT scans, which is very expensive, time-consuming, and even does the patient harm since both of them work with radiation  (Chatterjee et al., 2017). Hence, an urgent need for an artificial intelligence algorithm that could accurately detect chest-related diseases  (Islam et al., 2020; Salehinejad et al., 2018c; Salehinejad et al., 2018a).

Research done involved using deep learning to make predictions of medical images by extracting features from the images, including the shape and spatial rotation. Convolutional neural networks (CNNs) have played a very vital role in feature extraction and learning patterns that enabled prediction  (Demir, Sengur & Bajaj, 2020; Rustam et al., 2020; Toğaçar, Ergen & Cömert, 2020; Militante & Sibbaluca, 2020). For example, a CNN was used to improve extraction high-speed video endoscopy (HSV) when the training data is very little  (Wang et al., 2017).

AI is now mature to be embedded with the state of the art machine learning, and deep learning algorithm in different domains such as medicine  (Meraj et al., 2019), agriculture  (Rauf et al., 2019), biometrics  (Jin, Cruz & Goncalves, 2020), renewable energy  (Gao, Wang & Shen, 2020b), and cloud computing  (Gao, Wang & Shen, 2020c; Gao, Wang & Shen, 2020a). AI is gradually changing medicine as we know it, as more and more ways are being researched to automate medical practices and support the doctors and specialists  (Brag, 2020; Rauf et al., 2020; Kulkarni et al., 2020; Briganti & Le Moine, 2020). Many benefits are attached to artificial intelligence in medicine  (Jain et al., 2020). For example, rural areas and third-world countries usually have a scarcity of specialist doctors who could give them the care they need; artificial intelligence tools help them get the required medical assistance. Also, researches like this will help significantly in the health-care industry as specialists can use it to confirm their diagnosis; it can also be used by people who have little to no medical background. Even the patients could use this tool  (Mongan, Moy & Kahn, 2020; Ali et al., 2020; Tabares-Soto et al., 2020). All this is aimed at lessening the specialist’s burden since there is just a hand full of them, so they are focused only mainly on the X-rays that are flagged as suspicious by the tools used for the prediction. They also reduce doctors’ subjective opinions drastically, increase the speed of diagnosis, and accurately detect features that the human eye may ignore  (Zhang et al., 2020).

In this research, we employed deep neural networks to predict a wide range of chest-related diseases. Previous research used state-of-the-art techniques and got highly accurate results, but these were only able to detect one disease and were virtually useless for other diseases. The proposed methodology’s reliability and efficiency are slightly lower due to lack of data augmentation, highly normalized data, and ineffective hyperparameters. There is no practical way of accurately identifying multiple chest-related diseases from X-ray images with reliable results to the best of our knowledge.

The proposed deep learning models are employed to automatically detect the following chest-related diseases: Hernia, Pleural, Fibrosis, Emphysema, Pneumothorax, Pneumonia Atelectasis, Edema, Nodule, Mass, Infiltration, Effusion, Cardiomegaly and Consolidation and from the frontal view of chest X-ray images. Three different deep neural network architectures with large input types which have been pre-trained on extensive datasets are employed.

The objectives of the proposed research are as follow:

• To identify multiple chest-related diseases using deep CNNs and to tackle insufficient and unbalanced data through transfer learning.
• to employ synthetic data augmentation using deep CNNs for the detection of 14 chest-related diseases.
• To evaluate DenseNet121, InceptionResNetV2, and ResNet152V2 on multi-class identification problem.

## Related Work

Research done in medicine uses artificial intelligence to ease diagnosis, and some of them got beneficial and accurate results. Here we will discuss techniques used by previous researchers that used artificial intelligence to tackle chest-related diseases, using deep neural networks automatically. High-end medical infrastructures and qualified specialists are scarce, especially in rural areas and lower-middle-income countries (LMIC). Hence the medical teams available in these regions rely solely on CXRs for the detection of chest-related diseases. However, these CXRs are mainly used to diagnose pneumonia, which has to be done by a highly experienced radiologist as it is not an easy task  (Franquet, 2018).

### One-class detection of cardiothoracic diseases using deep neural networks

Sivasamy & Subashini (2020) used a Keras method to identify CXRs to predict lung diseases, and the model was 86.14 percent accurate, and it was then noted that the model’s accuracy increased as the number of training epochs raised.

Zhang et al. (2018) utilized pixel wisely transcribed DRRs data to learn a supervised multi-organ segmentation model in X-ray images the X-ray image structure, the gap in Nodule Annotations is challenging and time-consuming.

Rajpurkar et al. (2020) proposed a binary X-rays pneumonia detection classifier and obtained 0.435 f1 score. Salehinejad et al. (2018b) adopted a custom DCGAN model for X-ray image processing, equipped to produce artificial chest X-ray photographs. Since trained on an expanded dataset with DCGAN synthesized CXRs for real dataset balance (d3), the model has been incredibly accurate.

To classify pneumonia diseases, Chandra & Verma (2019) used five different models and got 95,631 percent as the highest accuracy. The model was restricted only to observation on automatically driven non-rigid deformable registration in lung regions and segmented feature extraction confined to ROI.

### Multi-class detection of cardiothoracic diseases using deep neural networks

Meta research was conducted in a few trials, which used state-of-the-art methods and had efficient results for one or two cardiothoracic diseases, but often they had the issue of misclassification. There are very few strategies that have targeted the 14 types of diseases associated with the chest.

To detect degenerated lung tissues in X-ray images, the authors used image descriptors  (Ke et al., 2019) dependent on Brightness, Hue’s spatial distribution values, and saturation in X-ray images, as well as a deep neural network in conjunction with Ant Lion and Moth-Flame algorithm. Based on the proposed fitness feature, the neural network analyses the object; however, if the probability of lung infections is identified, the hybrid method determines the morphed tissues in the X-ray image in detail.

Wang et al. (2019) provided the largest publicly available dataset on X-ray images used by the research community for experiments and training of models, and most of the research has been done showed promising results using deep convolutional neural networks. They further claimed that in order to add more disease names, the dataset could be expanded. A deep learning technique to classify fourteen underlying chest-related diseases was proposed by Rajpurkar et al. (2018). The proposed model was trained using a single X-ray image of the chest and generate the likelihood of each of the fourteen observations. They trained multiple models to see which one had the best performance, and in the end, DenseNet121 reached the best accuracy and was ultimately used for the testing, but it was limited to the CheXpert database and prone to over-fitting.

The authors suggested a hybrid approach  (Sahlol et al., 2020) for classifying chest X-ray images efficiently. MobileNet, in combination with transfer learning based on a CNN model initially trained on the ImageNet dataset, is used to extract features from chest X-ray images. They used the AEO meta-heuristic algorithm as a component predictor to decide whichever of these attributes seem to be the most important. The proposed approach is tested against two publicly available benchmark datasets, Dataset 2 and Shenzhen. It enables them to obtain significant efficiency while cutting down on computing time.

Ho & Gwak (2019) used a pre-trained DenseNet121 model with feature extraction techniques to identify fourteen chest-related diseases accurately. Description of some related work indicating pros and cons of existing approaches are given in Table 1.

## Materiel and Methods

### ResNet (Residual Neural Network)

The ResNet model uses residual sharing, and the training process becomes more tedious and complicated as the depth of the network increases, the convergence time also increases significantly. The deep neural networks are exposed to degradation when the network starts the convergence process (Kao et al., 2011). For example, a deep neural network’s accuracy starts to degrade rapidly when the network becomes stagnant due to saturation. This research aims at making use of residual mapping, which will fit the layers of the neural network, and this makes ResNet a perfect choice of a model for trial as it has sufficient accuracy and the depth of the model be adjusted.

**Table 1:** Description of some related work indicating pros and cons of existing approaches.

Ref	Data set	Method	pros	cons
Pereira et al. (2020)	RYDLS-20	Early and late fusion techniques	Multi-class classification+ hierarchical classification	Data sparsity and feature optimization missing
Apostolopoulos, Aznaouridis & Tzani (2020)	Custom	Mobile Net	Considered feature extraction approaches	6-class problem
Oh, Park & Ye (2020)	Custom	Patch-based CNN	Employed Potential imaging biomarkers for validation	5-class problem
Apostolopoulos & Mpesiana (2020)	Custom	Transfer learning based CNN	Considered bacterial and viral pneumonia target class	5-class problem
Yoo et al. (2020)	Custom	Decision-tree classifier	Merging deep learning in tree classifier	3-class problem

### DenseNet (Densely Connected Convolutional Networks)

DenseNet is a neural network used for visual object recognition, and it is quite similar to ResNet with a few fundamental differences. The traditional convolutional neural network architecture with L layers has L connections, and these connections are feed-forward, which carry information from one layer to the contagious layer. However, in Wang et al. (2019), the authors suggest that there are direct (L(L+1)/2) links to a deep convolutional neural network, implying that previous layer maps for a coevolutionary neural network serve as input to the current layer. DenseNet provides densely connected front and back layers that let the context spread throughout the workout process, the main difference between Resnet and DenseNet. DenseNet also allows the reuse of features that channel connexions.

Another distinction is that ResNet uses the additive approach (+) that combines the previous layer with the next layer, while DenseNet concatenates the previous layer’s output layer with the next layer. It was discovered that DenseNet could successfully reduce the number of training parameters according to the experiments in Wang et al. (2019). It also reduces the problem of vanishing gradient, strengthening the feature propagation and effective reuse of features.

### InceptionResNetV2

InceptionResNetV2 is a new and more accurate model based on CNN architecture validated accuracy on the ImageNet database with more than a million images. It is a variation of the InceptionV3 (Szegedy et al., 2016) and has 164 layers that use residual connections in the Inception architecture.

### Model architecture

A convolutional neural network is a series of deep networks where consecutive layers receive input from the layers before them and pass output to the next layer; this continues until the final layer. The amount of deep networks is proportional to the prediction’s strength and efficiency, meaning models with deeper networks tend to perform better than models with external networks (Szegedy et al., 2016). The reliability of the model also depends on the degree of approximation, where deeper abstractions would describe the various oriented boundaries of the image, medium abstraction levels may describe the portions of an image, and larger object parts and the object as a whole are represented in higher levels. In this research, we experiment on deep neural networks’ capabilities in the automatic detection of cases in chest pathology.

**Figure 1:**
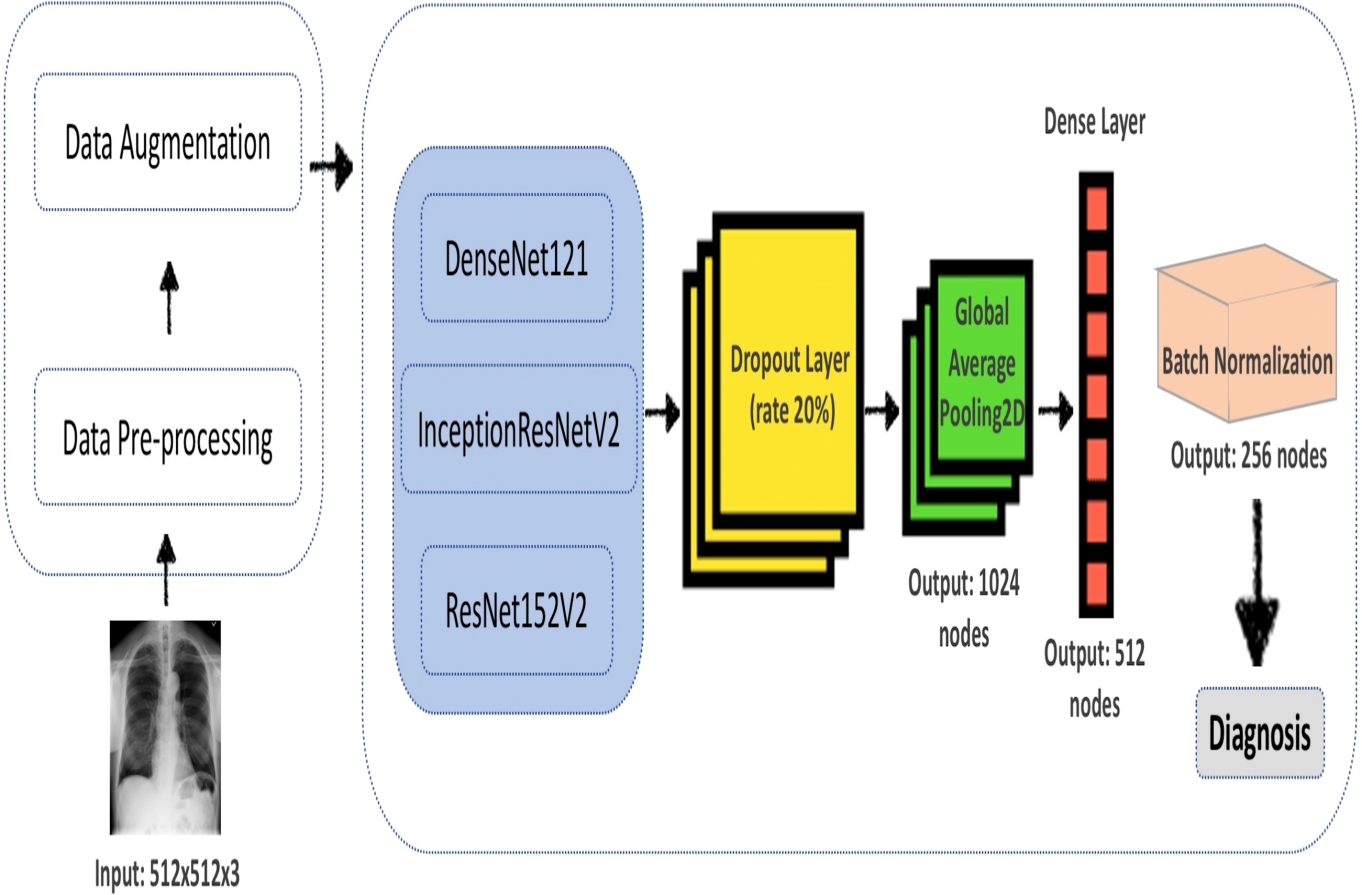
Overview of the proposed work framework with three deep convolutional neural networks models.

For this research, three deep convolutional neural networks are experimented on. Figure 1 depicts our proposed framework, representing the conceptual process of different deep learning model architectures used in this research. TensorFlow, which Google develops, is the leading deep learning framework used throughout the development process to create, train, validate, fine-tune and test different architectures. Apart from TensorFlow, Pandas has been used for data analysis and matplotlib for creating a visualization. The training data is first loaded into the workspace and the corresponding labels using panda’s data frame. There are a total of 14 pathological disease labels that the entire dataset has been based on.

**Figure 2:**
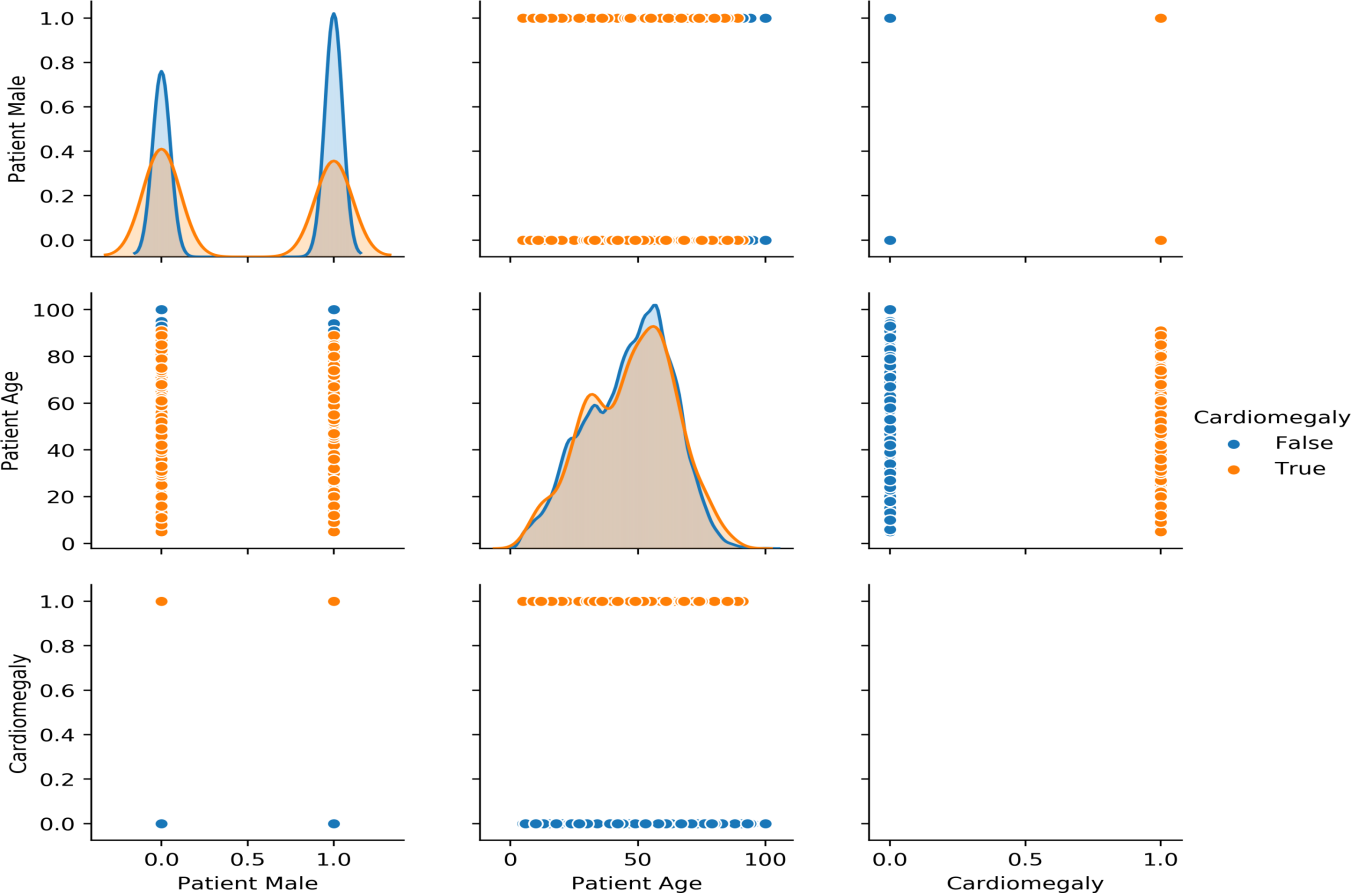
Gender and Age wise distribution of chest related diseases.

Figure 2 shows the gender and age-wise distribution of diseases. It shows several patients of each gender concerning age. It can be observed that Cardiomegaly affects people starting around the age of 10 upwards but most common between the age of 35 to 60, with the median age around 50. It can also be seen that the male gender is more affected than the female by most of the diseases.

## Experimental Study and Analysis

### Dataset preparation and preprocessing

**Figure 3:**
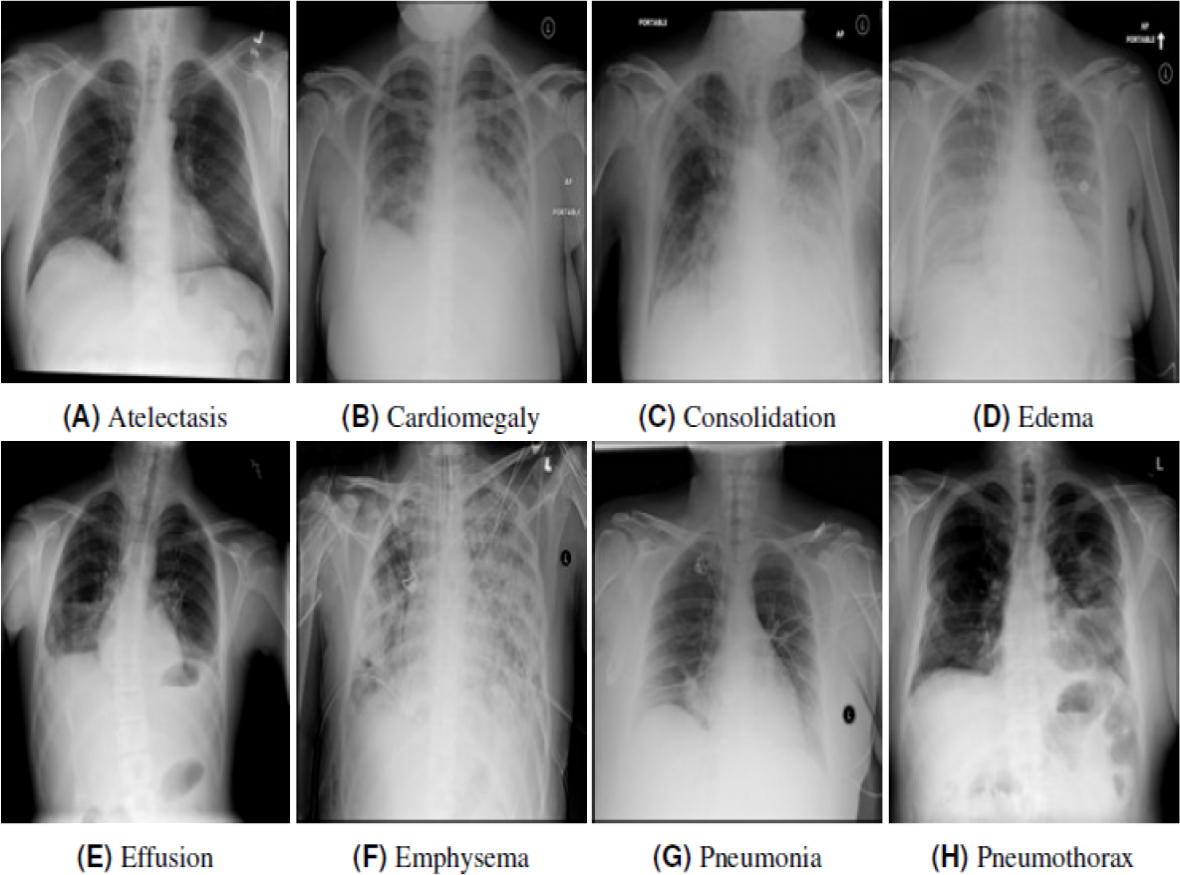
Chest X-rays categories pathology with related labels in Chest X-ray14. Each image is labelled with one pathology. (A) Atelectasis, (B) cardiomegaly, (C) consolidation, (D) edema, (E) effusion, (F) emphysema, (G) pneumonia, (H) phenothorax.

This analysis uses a detailed state-of-the-art datasetcollected from Wang et al. (2019). A total of 112,120 frontal-view chest X-rays (CXRs) images collected from 30,805 unique patients are included in the dataset. All the images were labeled with one of the 14 pathological diseases and classified as “No Findings” for those with no disease. The Hernia class eventually withdrew from the label, leaving 13 main classes because the images were smaller than the others. Some examples and their corresponding annotations in Chest X-ray14 (Wang et al., 2019) are shown in Fig. 3.

One of the significant problems faced with this research is that the classes’ images are not enough for proper training of our models; hence, to get effective results with high accuracy, we generalized the data by considering data augmentation. Image augmentation provides training images by alternate processing or assembly methods of many systems, such as random rotation, turns, shear, flips, zooming, etc.

Next, as part of the data pre-processing, a folder was created for each of the class labels, and each folder was further subdivided into training and validation sets. The training and validation folders contained the class label as a sub-folder, enabling the model to read the data accurately. Before the model’s training began, the training and validation datasets were reshaped to (150, 150, 3), and they were normalized, then image augmentation was applied with a certain degree of rotation on the training dataset. Finally, using one-hot encoding, the class labels were encoded, and the array was reshaped to (128, 128, 3), which were stored as pickle objects. It was not easy to recognize any patterns that divided the images into their different groups when these images were observed.

### Training methodology

This section explains the training procedure undertaken to train different deep learning model architectures. TensorFlow is the leading deep learning framework used throughout the development process to create, train, validate, fine-tune and test different architectures. Apart from TensorFlow, Pandas has been used for data analysis and matplotlib for creating a visualization. The training data is first loaded into the workspace and the corresponding labels using panda’s data frame. There are a total of 14 pathological disease labels that the entire dataset has been based on.

### Training process

1. The model of choice, as seen in Fig. 1, is first loaded into a workspace with a pre-trained weight set to the ‘ImageNet’ dataset. To add the classifying layer to this model’s head, a GlobalAveragePooling2D layer is added at the end. Then the output of this layer is flattened, and added to it is another dense layer of 512 activation units using ‘ReLU’ as the activation function, finally. An output layer of 14 activation units is added to complete the model corresponding to the 14 labels with activation function as ‘sigmoid.’
2. To add image augmentation to the dataset, the following *ImagDataGenerator* has been added: * core_idg = ImageDataGenerator(rescale=1 / 255, samplewise_center=True, samplewise_std_normalization=True, horizontal_flip=True, vertical_flip=False, height_shift_range=0.05, width_shift_range=0.1, rotation_range=10, shear_range=0.1, fill_mode=’nearest’, zoom_range=0.15)* This mainly rescales the images and horizontal flips the images and few other functionalities like shift range, zooming, rotation, etc.
3. To compile the model, ‘*binary_crossentropy*’ has been used as a loss function and ‘Adam’ as the main optimizing algorithm with ‘accuracy’ as a metric.
4. The training starts with a learning rate of 0.001 but is eventually lowered using *ReduceLROnPlateau* scheduler till it reaches 0.00001.
5. The model is initially trained with all the layers of the pre-trained model set to freeze for 30-40 epochs, then to further fine-tune the model, a second training phase is initiated with the same number of epochs, but this time is training all the layers of the model including the pre-trained weights from *ImageNet*’. This further helps to improve our accuracy and transforms our model to generalize better on our X-ray dataset.

## Results

In the present study, we investigate three deep convolutional neural network models (DenseNet121, InceptionResNetV2, and ResNet152V2) to design the predictive model. Later, we compare the first two models to show competitiveness compared to another model called ResNet152V2. Thus, we later focused our analysis based on DenseNet121 and InceptionResNetV2 models designed and tasked with classifying X-ray images of chest-related diseases for the final prediction. Every neural network has a 150 × 150 × 3 input structure. All three networks are built either with or without available data for each of the simulations’ models. We hardly have used orientation parameters to increase data, which produces data through a rotation of 10-degree angles of the images. The data was distributed as 80% and 20% for training and testing, respectively.

The hyperparameters of the models are learning rate (0.001), batch size (32), Softmax and ReLU, respectively, and epochs (40) for DenseNet121 and epochs (30) for InceptionResNetV2.

The predictive model is trained by the state-of-the-art training datasets collected by Wang et al. (2019). A summary of the comparison between actual and predicted classes is provided in Table 2. The accuracy between the actual and predicted classes shown in Table 2 presents the relevance in evaluating classification performance. It shows the result’s high accuracy and minimizes the errors in between predictive and actual data sets.

**Table 2:** A comparison of actual and predicted accuracies of DenseNet121 and InceptionResNetV2.

**Pathology**	**Model**	**Actual**	**Predicted**
Atelectasis	DenseNet121	20.21%	22.10%
	InceptionResNetV2	20.51%	21.57%
Cardiomegaly	DenseNet121	4.79%	5.71%
	InceptionResNetV2	6.15%	5.63%
Consolidation	DenseNet121	9.86%	8.56%
	InceptionResNetV2	8.30%	8.88%
Edema	DenseNet121	4.49%	4.39%
	InceptionResNetV2	4.10%	4.67%
Effusion	DenseNet121	25.88%	28.07%
	InceptionResNetV2	26.07%	25.39%
Emphysema	DenseNet121	4.79%	3.59%
	InceptionResNetV2	4.49%	4.04%
Fibrosis	DenseNet121	3.03%	2.56%
	InceptionResNetV2	2.44%	3.26%
Infiltration	DenseNet121	40.14%	37.92%
	InceptionResNetV2	40.53%	39.67%
Mass	DenseNet121	12.30%	11.13%
	InceptionResNetV2	13.28%	11.30%
Nodule	DenseNet121	11.91%	11.28%
	InceptionResNetV2	12.70%	12.21%
Pleural_Thickening	DenseNet121	7.23%	6.34%
	InceptionResNetV2	7.03%	6.20%
Pneumonia	DenseNet121	3.22%	2.56%
	InceptionResNetV2	2.83%	2.57%
Pneumothorax	DenseNet121	8.89%	8.84%
	InceptionResNetV2	9.77%	9.83%

The loss is a quantitative measure of how far from the actual output the prediction made; this means that models with little losses make more accurate predictions than the models with large losses. The loss is calculated for both the training and validation to see how well the model is working and ensure no problems of under-fitting or over-fitting.

An epoch is a term in deep learning to show the number of times the whole training dataset is utilized once to update the weights (Albahli, 2020). It is chosen by the network designer so that the loss is minimal and does not increase, and since loss and accuracy are inversely proportional, at the point where loss is minimum, the accuracy will be at its highest point. We set our epochs to 40 because both models reached a stable low loss when the epochs were less than or equal to 40.

Table 3 shows the comparison of the training and validation for both loss and accuracy of all the three convolutional neural networks that were initially used in this research. InceptionResNetV2 model got an average Area Under the Receiver Operating Characteristic Curve (ROC-AUC) score of 0.801 when detecting the pathological diseases.

**Table 3:** The proposed models and comparisons of their results.

**Models**	**Train_loss**	**Train_accuracy**	**Val_loss**	**Val_accuracy**	**ROC-AUC score**
DenseNet121	0.2596	0.4238	0.2645	0.4043	0.793
InceptionResNetV2	0.2446	0.4547	0.2641	0.4102	0.801
ResNet152V2	0.2827	0.3686	0.2821	0.3760	0.751

**Figure 4:**
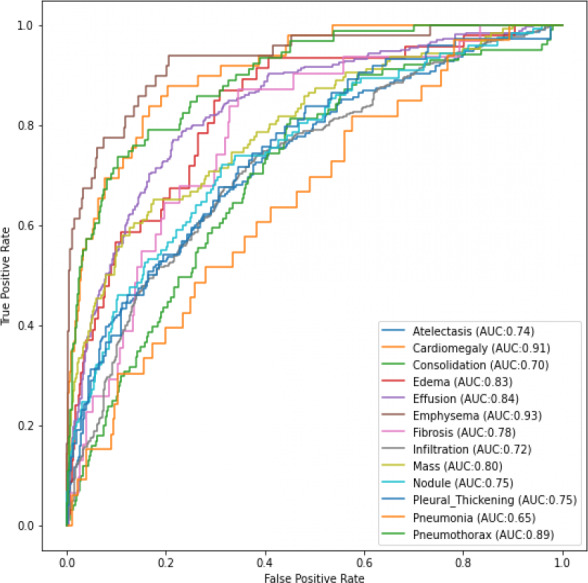
ROC AUC scores for DenseNet121 of the 13th classes of chest-related diseases.

**Figure 5:**
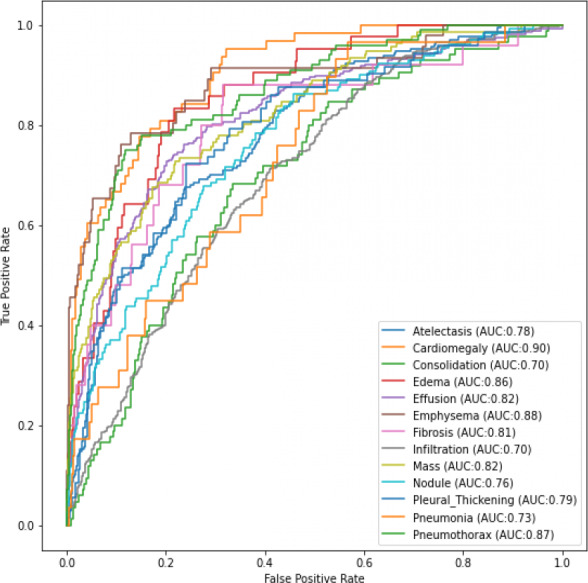
ROC AUC scores for InceptionResNetV2 of the 13th classes of chest-related diseases.

Figures 4 and 5 shows the ROC-AUC graphs for the DenseNet121 and InceptionResNetV2 respectively. The curves have two parameters plotted: True Positive Rate (TPR) and False Positive Rate (FPR). When we compare the two models, it is seen that InceptionResNetV2 got a higher score for the number of classes predicted. This makes the InceptionResNetV2 better than the other two deep learning models used in this research, as seen in Table 3. It can also be seen that the ResNet152V2 had the lowest performance when compared to the other two in terms of training and validation accuracies when data augmentation was used. However, in terms of the loss, Table 3 shows the training and validation losses instead of the training and validation accuracy.

From the results obtained, it can be seen that InceptionResNetV2 and DenseNet121 are more efficient for the classification task using data augmentation. It can also be seen that InceptionResetV2 and DenseNet121 have an 80% improvement in the AUC score over the ResNet152V2 model. There is also a small gap between the training and validation results regarding accuracy and loss. As more data is gotten, there is a very high possibility of the current performing better, and different models could also give highly effective results.

To sum up, Table 4 shows a comparison between our DenseNet121 and InceptionResNetV2 model with similar researches done in the field of chest pathology using deep learning. It can be seen that of the 14 cardiothoracic diseases that were tested for, our models got a much higher accuracy in 13 of them, except for Consolidation. It should be noted that Gundel et al. (2019) trained their model with 180,000 images from the PLCO dataset (Ho & Gwak, 2019) as extra training data.

**Table 4:** AUC scores comparisons of the 13th chest-related diseases.

Pathology	DensNet	InceptionResNetV2	Wang et al.	Yao et al. (2017)	Wang & Xia (2018)	Gundel et al.
Atelectasis	0.74	0.78	0.71	0.77	0.74	0.76
Cardiomegaly	0.91	0.90	0.80	0.90	0.87	0.88
Consolidation	0.70	0.70	0.70	0.78	0.72	0.74
Edema	0.83	0.86	0.83	0.88	0.83	0.83
Effusion	0.84	0.82	0.78	0.85	0.81	0.82
Emphysema	0.93	0.88	0.81	0.82	0.82	0.89
Fibrosis	0.78	0.81	0.76	0.76	0.80	0.80
Infiltration	0.72	0.70	0.60	0.69	0.67	0.70
Mass	0.80	0.82	0.70	0.79	0.78	0.82
Nodule	0.75	0.76	0.67	0.71	0.69	0.75
Pleural_Thickening	0.75	0.79	0.70	0.76	0.75	0.76
Pneumonia	0.65	0.73	0.63	0.71	0.69	0.73
Pneumothorax	0.89	0.87	0.80	0.84	0.81	0.84

### Limitation of proposed framework

In DenseNet121, we utilize L-1 total layers for replacing the convolution operation with a CNN layer and a transformation up average pooling operation. An extrapolated convolution layer uses the previous function to maps in the next pooling layer. To form a new dense block’s input, the input images feature maps are concatenated with the skip relation ones. Since the fully connected layers raise the dynamic range of the feature maps, exponential growth in the range of features would have been too storage-intensive, particularly for the pre-softmax layer’s full resolution features. The proposed study’s order limitation is the lack of training parameters in InceptionResNetV2, which is tackle by adopting transfer learning.

## Conclusion

Diagnosis of chest-related diseases from X-ray images is a challenging task that requires special attention from the research community because of the rising rate at which the diseases are contracted. However, there are a few types of research done related to chest-related diseases. All the previous research has been done in chest-related diseases centered around only one class disease, but research is multi-class and accurately investigated 14 classes of chest-related diseases with reliable results. This research used three deep neural network architectures; DenseNet121, InceptionResNetV2, and ResNet152V2, and we were able to get a beneficial result in detecting chest-related diseases from X-ray images. InceptionResNetV2 got the highest ROC-AUC score in the three models we used for this research. The major limitation of this research is that all the images used for both training and validation are images of the chest’s frontal view. However, specialists say literal view yields more accurate results. Hence, for future works, researches will be done using literal views of the chest X-rays, and the results will be compared.

## Additional Information and Declarations

### Data Availability

The following information was supplied regarding data availability: Data is publicly available at Kaggle https://www.kaggle.com/nih-chest-xrays/data.
